# Peroxisome degradation in mammals: mechanisms of action, recent advances, and perspectives

**DOI:** 10.3389/fphys.2013.00145

**Published:** 2013-06-14

**Authors:** Marcus Nordgren, Bo Wang, Oksana Apanasets, Marc Fransen

**Affiliations:** Laboratory of Lipid Biochemistry and Protein Interactions, Department of Cellular and Molecular Medicine, Katholieke Universiteit LeuvenLeuven, Vlaams-Brabant, Belgium

**Keywords:** peroxisomes, organelle quality control, reactive oxygen species, protein import, organelle turnover, autophagy, pexophagy, lysosomes

## Abstract

Peroxisomes are remarkably dynamic organelles that participate in a diverse array of cellular processes, including the metabolism of lipids and reactive oxygen species. In order to regulate peroxisome function in response to changing nutritional and environmental stimuli, new organelles need to be formed and superfluous and dysfunctional organelles have to be selectively removed. Disturbances in any of these processes have been associated with the etiology and progression of various congenital neurodegenerative and age-related human disorders. The aim of this review is to critically explore our current knowledge of how peroxisomes are degraded in mammalian cells and how defects in this process may contribute to human disease. Some of the key issues highlighted include the current concepts of peroxisome removal, the peroxisome quality control mechanisms, the initial triggers for peroxisome degradation, the factors for dysfunctional peroxisome recognition, and the regulation of peroxisome homeostasis. We also dissect the functional and mechanistic relationship between different forms of selective organelle degradation and consider how lysosomal dysfunction may lead to defects in peroxisome turnover. In addition, we draw lessons from studies on other organisms and extrapolate this knowledge to mammals. Finally, we discuss the potential pathological implications of dysfunctional peroxisome degradation for human health.

## Introduction

Peroxisomes were first observed in electron microscopy studies by the Swedish doctoral student Johannes Rhodin in 1954 (Rhodin, 1954) and, approximately a decade later, for the first time isolated from rat liver and biochemically characterized by the Belgian Nobel Laureate Christian de Duve and his colleague Pierre Baudhuin (de Duve and Baudhuin, 1966). The name “peroxisome” derives from the early observation that the organelle is involved in processes that both generate and decompose hydrogen peroxide (H_2_O_2_). Over the last half century, our knowledge about this highly dynamic and plastic organelle has virtually exploded. For example, it is now known that mammalian peroxisomes are involved in multiple metabolic pathways, including the breakdown of various carboxylates via α- and β-oxidation, and the synthesis of bile acids, docosahexaenoic acid (DHA) and ether-phospholipids (Van Veldhoven, 2010). Importantly, many of the enzymes involved in these processes produce reactive oxygen or nitrogen species (ROS or RNS) as part of their normal catalytic cycle (Fransen et al., 2012). To combat the destructive effects of these molecules, peroxisomes also contain various antioxidant enzymes of which catalase is perhaps the best known (Antonenkov et al., 2010). The necessity of peroxisomes for normal development and physiology is illustrated by the existence of a group of genetic disorders associated with peroxisomal deficiencies. These diseases are generally subdivided into two groups: the peroxisome biogenesis disorders (PBDs) (Nagotu et al., 2012) and the single peroxisomal enzyme deficiencies (PEDs) (Wanders and Waterham, 2006). In recent years, peroxisome (dys)function has also been associated with a wide variety of age-related maladies, including cancer, type 2 diabetes, and neurodegeneration (Fransen et al., 2013).

## Physiological importance of peroxisome homeostasis

Currently, it is generally accepted that the localization and activity of many proteins (e.g., kinases, phosphatases, transcription factors, etc.) are reversibly controlled by the cellular composition and concentration of specific lipids and (redox-derived) signaling mediators (Hekimi et al., 2011; Schug et al., 2012). As peroxisomes are actively involved in the metabolism of many of these compounds, it is not surprising that these organelles are increasingly recognized as potential signaling platforms in diverse biological processes such as inflammation (Zmijewski et al., 2009), apoptosis (Li et al., 2002; Hasegawa et al., 2010), innate immunity (Dixit et al., 2010; Horner et al., 2011), cellular aging (Beach et al., 2012; Giordano and Terlecky, 2012), diabetes (Elsner et al., 2011; Hwang et al., 2012), and cancer development (Reddy et al., 1980; Frederiks et al., 2010). This is perhaps best illustrated by the observation that peroxisomes play a central role in the cellular metabolism of H_2_O_2_, a key molecule in cellular redox signaling (Fransen et al., 2012). For example, peroxisomes seem to be responsible for as much as 35% of the total H_2_O_2_ production in rat liver (Boveris et al., 1972), and fibroblasts derived from hypocatalasemic patients accumulate H_2_O_2_ and are oxidatively damaged (Wood et al., 2006). In addition, overexpression of acyl-CoA oxidase 1, a H_2_O_2_-producing enzyme of the peroxisomal fatty acid β-oxidation pathway, has been shown to activate the redox-sensitive transcription factor NF-κB in a substrate concentration-dependent manner (Li et al., 2000); and overexpression of catalase, a peroxisomal enzyme that decomposes H_2_O_2_, sensitizes cells (and animals) to certain types of stressors by dampening H_2_O_2_-mediated signaling pathways (Carter et al., 2004; Chen et al., 2004). Finally, as high ROS levels are also known to cause significant damage to cell structures (Nathan and Ding, 2010), excessive production of peroxisomal ROS may overwhelm the cellular antioxidant defenses and mediate cellular injury or even cell death (Elsner et al., 2011; our unpublished observations). In this context, it is also interesting to mention that, to carry out their functions, peroxisomes physically and functionally interact with other cell organelles (Horner et al., 2011; Beach et al., 2012; Kohlwein et al., 2013), and that disturbances in peroxisome function have been reported to trigger signaling events that ultimately activate mitochondrial and endoplasmic reticulum stress pathways (Koepke et al., 2007; Ivashchenko et al., 2011; Kovacs et al., 2012). In summary, these observations (among others) clearly illustrate that changes in peroxisomal metabolism have a tremendous impact on many cellular processes, and as such it is of vital importance for humans (and organisms in general) to adjust peroxisome function and abundance to cellular needs.

## Regulation of peroxisome abundance

**Figure 1:**
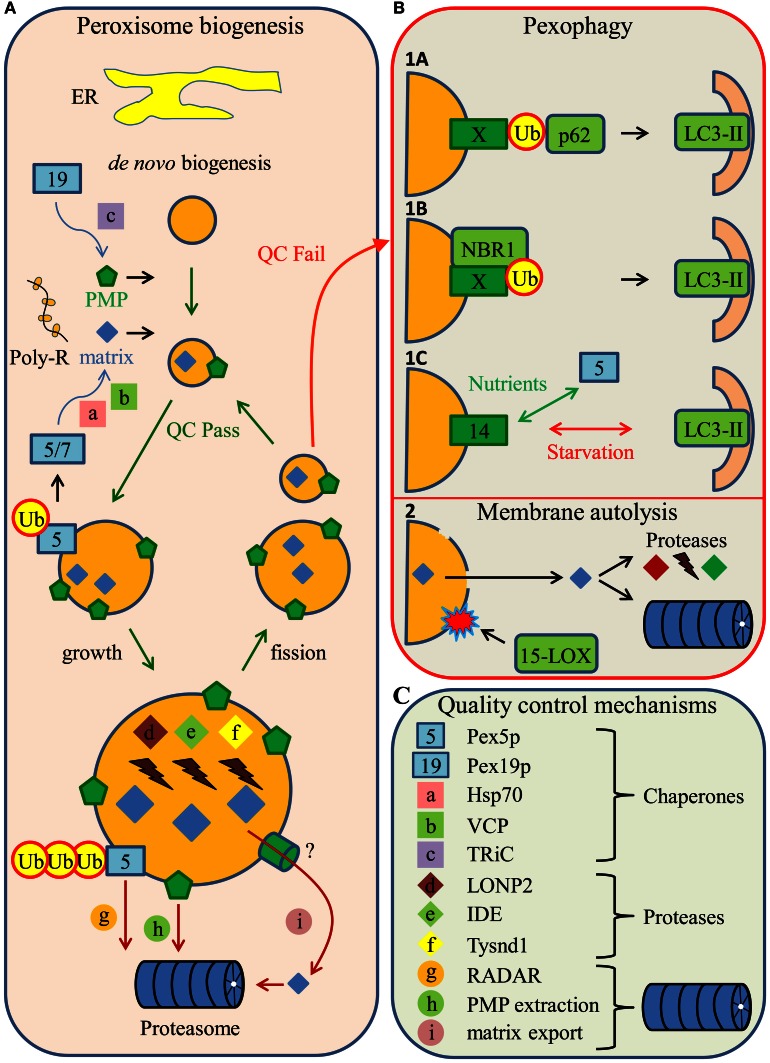
**Peroxisome biogenesis, quality control, and turnover in mammalian cells. (A)** Peroxisomes can be formed *de novo* from the ER or by growth and asymmetric fission of pre-existing organelles. Peroxisomal matrix (matrix) and membrane (PMP) proteins are translated on free polyribosomes (Poly-R) in the cytosol, where they are recognized by their cognate import receptors Pex5p, Pex7p, or Pex19p (these and other peroxins are represented by numbers). Importantly, Pex5p and Pex19p possess chaperone-like activities. In addition, matrix protein folding is facilitated by the cytosolic chaperones Hsp70 and VCP, whereas PMP folding is assisted by the chaperonin TRiC (all non-peroxin-related quality control mechanisms are indicated by lower-case letters and listed in panel **C**). At the peroxisomal membrane, Pex5p is either mono- or poly-ubiquitinated (Ub). In the case of mono-ubiquitination, Pex5p is extracted from the membrane into the cytosol for a new round of matrix protein import. However, upon poly-ubiquitination, Pex5p is degraded by the proteasome in a process known as RADAR. Superfluous or dysfunctional PMPs are also targets for proteasomal degradation. The peroxisomal matrix harbors several proteases (e.g., LONP2, IDE, and Tysnd1) that function as regulators of intra-peroxisomal proteostasis. In addition, excessive peroxisomal matrix proteins may be exported to the cytosol where they are degraded by cytosolic proteases or the proteasome. During their life cycle, peroxisomes are constantly exposed to quality control (QC) mechanisms, and in case of failure, it is likely that the organelle is targeted for degradation. **(B)** Mammalian peroxisomes can be degraded by distinct pathways, pexophagy and 15-LOX mediated membrane autolysis. Three mechanisms have been proposed for how dysfunctional peroxisomes can be removed by the autophagic machinery. **(1A)** The first one involves the recognition of a ubiquitinated PMP (X) by an autophagic adaptor protein p62 which, in turn, bridges the peroxisome with the developing autophagosome via LC3-II. **(1B)** The second mechanism involves another adaptor protein, NBR1, which, similarly to p62, recognizes dysfunctional peroxisomes via ubiquitinated PMPs and links the organelles with the autophagic machinery through LC3-II. NBR1 is also capable of binding directly to the peroxisomal lipid bilayer. **(1C)** A third mechanism describes the competitive nature of the Pex14p binding partners Pex5p and LC3-II. Under nutrient-rich conditions, Pex5p is the preferred binding partner, whereas in nutrient-starved conditions, interaction with LC3-II is favored. Importantly, peroxisomes are only degraded upon re-supplementation of nutrients. **(2)** Finally, the peroxisomal membrane can undergo 15-lipoxygenase (15-LOX)-mediated autolysis, which subsequently leads to proteasome- or protease-dependent degradation of peroxisomal proteins. **(C)** Peroxisomal protein quality control mechanisms.

Peroxisome abundance is strictly regulated by the rates of organelle formation, division and turnover. Peroxisomes can be formed either *de novo* from the ER or by growth and asymmetric division of pre-existing organelles (Figure 1A) (Fransen, 2012). The latter process is, to a great extent, regulated by the Pex11p family of proteins. Indeed, the expression levels of members of this protein family have been shown to correlate with the number of peroxisomes in a cell (Schrader et al., 1998; Thoms and Erdmann, 2005), and overexpression of human Pex11pβ promotes peroxisome proliferation independent of peroxisomal metabolic activity (Li and Gould, 2002). For more detailed information regarding these processes, we refer the reader to other recent reviews (Ma et al., 2011; Schrader et al., 2012).

As mentioned above, peroxisomes can rapidly modulate their number, size, and function in response to cellular needs. Nowhere else is this better illustrated than in the methylotrophic yeasts *Hansenula polymorpha* and *Pichia pastoris*, where peroxisome number and size are massively increased when the cells are grown in media containing methanol as the sole carbon source (van der Klei and Veenhuis, 2006). This finding may not be surprising given that these organelles harbor the key enzymes of methanol metabolism (van der Klei and Veenhuis, 2006). As the enhanced peroxisomal activity is no longer needed when the cells are recultivated in media containing ethanol or glucose as carbon source, these methanol-induced peroxisomes are rapidly degraded by a process called “pexophagy” (see below) (Manjithaya et al., 2010). A similar phenomenon, albeit less pronounced, can also be observed in rodents upon the administration (and subsequent removal) of a variety of xenobiotics, collectively known as peroxisome proliferators (Reddy et al., 1980; Yokota, 1993). Agents that are frequently used to induce peroxisome proliferation in this class of animals include hypolipidemic drugs (e.g., fibrates), industrial phthalate ester plasticizers, and several types of fatty acids (Cho et al., 2008). These compounds act by binding to the nuclear receptor Peroxisome Proliferator-Activated Receptor α (PPARα) (Issemann and Green, 1990), which heterodimerizes with the Retinoid X Receptor (RXR) to regulate gene expression through PPAR-responsive elements in target DNA (Chandra et al., 2008). Interestingly, human cells do not respond similarly to PPARα agonists (Lawrence et al., 2001). However, some drugs such as 4-phenylbutyrate and niclosamide can act as potent PPARα-independent peroxisome proliferators in these cells (Sexton et al., 2010).

It is well-known that peroxisome number is significantly reduced in fibroblasts from patients with PBDs or peroxisomal fatty acid β-oxidation deficiencies (Chang et al., 1999). Intriguingly, in fibroblasts from the latter class of patients, this decrease in number coincides with an increase in peroxisome diameter but has apparently no effect on the expression levels of peroxisomal membrane proteins (PMPs) (Chang et al., 1999; and references therein). Together with the observations that (1) the reduced abundance of peroxisomes in cells with peroxisomal β-oxidation deficiency correlates with a loss of the corresponding enzyme activity and not with peroxisomal import defects (Chang et al., 1999), and (2) overexpression of ACOT8, one of the peroxisomal acyl-CoA thioesterases that inhibit fatty acid oxidation by depleting acyl-CoA substrates, reduces peroxisome abundance in normal human fibroblasts (Chang et al., 1999), these data suggest that this dysmorphogenesis is caused by alterations in peroxisomal β-oxidation metabolite levels. This hypothesis is in line with the findings of a recent study (Itoyama et al., 2012) showing that treating cells with DHA, a major product of peroxisomal β-oxidation, restores peroxisome number in cells deficient in peroxisomal β-oxidation, but not in PBD cells. Importantly, this process is time-, dose-, and Pex11p-dependent, but PPARα-independent. As peroxisomes in control fibroblasts fail to proliferate in response to DHA treatment, these findings also underscore the complexity of the regulation of peroxisome abundance under normal conditions.

## Peroxisome quality control mechanisms

To maintain their health, cells need to keep organelles in a functional state. Over the years, multiple quality control mechanisms have been described, including (1) organellar chaperones and proteases that, respectively, promote proper protein folding and proteolytic removal of terminally damaged proteins (Haynes and Ron, 2010; Walter and Ron, 2011), (2) retrotranslocation of misfolded proteins from the organelle to the cytosol for proteasomal degradation (Taylor and Rutter, 2011; Brodsky, 2012), and (3) autophagic degradation of dysfunctional organelles (Farré et al., 2009). A similar situation most likely exists for peroxisomes. In the following paragraphs, we discuss the components and mechanisms involved in peroxisomal proteostasis. For the mechanisms of how dysfunctional peroxisomes are degraded by the autophagic machinery, please see section Peroxisome Degradation of this chapter below.

The involvement of chaperones and proteases is central to many organellar quality control systems (Chen et al., 2011). Indeed, newly imported proteins often need to be proteolytically processed, properly folded, and assembled into functional units to acquire their activity. In this context, it is important to note that peroxisomes have the capacity to import fully folded and oligomeric matrix proteins (Lanyon-Hogg et al., 2010). This finding suggests that the quality control of proteins destined for the peroxisomal matrix may occur, at least partially, in the cytosol. Such control mechanisms may be mediated by cytosolic heat shock proteins (Hsps) or cytosolically located peroxins (proteins involved in peroxisome biogenesis) displaying chaperone-like activity. Potential candidates include members of the Hsp70 family of proteins (Walton et al., 1994; Harano et al., 2001), valosin-containing protein (Murakami et al., 2013), and Pex5p, the import receptor for peroxisomal matrix proteins containing a C-terminal peroxisomal targeting signal (PTS1) (Figure 1) (Freitas et al., 2011). Importantly, as the peroxisomal matrix protein translocation machinery can also accommodate the import of unfolded proteins (Brocard et al., 2003), one would expect peroxisomes to contain classical Hsps (e.g., members of the Hsp70 superfamily). Here it should be mentioned that Hsc70 molecules can be co-imported into peroxisomes by interacting with unfolded PTS1-bearing albumin (Brocard et al., 2003). In addition, one cannot exclude the possibility that the peroxisomal matrix harbors other proteins having a chaperone-like activity. One such protein may be the peroxisomal Lon protease (see below), of which the *Penicillium chrysogenum* orthologue has been shown to possess chaperone activity *in vitro* (Bartoszewska et al., 2012). Finally, peroxisome formation and maintenance also require the proper assembly of membrane proteins. In this context, it should be emphasized that Pex19p, the cycling import receptor for newly synthesized PMPs, also exhibits chaperone-like activity (Figure 1) (Jones et al., 2004). In addition, it has been reported that *in vitro* translated PMP22 forms a complex with TRiC (Figure 1) (Pause et al., 1997), a cytosolic chaperonin known to fold a large number of protein substrates (Spiess et al., 2006).

Several proteins in the peroxisomal matrix are post-translationally processed by specific proteases (Okumoto et al., 2011). In addition, as peroxisomes constantly produce ROS (Fransen et al., 2012), the presence of sophisticated intra-peroxisomal quality control mechanisms is essential. Damaged, oxidized and misfolded proteins need to be degraded in order to maintain peroxisome proteostasis and function. To date, three proteases have been identified in mammalian peroxisomes, including insulin degrading enzyme (IDE) (Authier et al., 1994), peroxisomal Lon protease (LONP2) (Kikuchi et al., 2004), and trypsin domain-containing protein 1 (Tysnd1) (Figure 1) (Kurochkin et al., 2007). IDE has been shown to degrade the cleaved leader peptide of the peroxisomal enzyme thiolase as well as oxidized lysozyme, a model substrate for oxidized proteins (Authier et al., 1994; Morita et al., 2000). LONP2 is a multi-functional protein that has chaperone-like functions (see above) and displays proteolytic activity toward (superfluous) β-oxidation enzymes (Yokota et al., 2008; Okumoto et al., 2011). The *P. chrysogenum* orthologue of this protein can degrade oxidized proteins *in vitro*, and an inactivation of its function has been shown to be associated with the formation of protein aggregates in the peroxisomal matrix and enhanced oxidative stress (Bartoszewska et al., 2012). In this context, it is important to note that LONP1, the mitochondrial Lon protease, is the most important quality control protease in the mitochondrial matrix, where it selectively degrades damaged, unassembled and misfolded proteins (Venkatesh et al., 2012). Finally, Tysnd1 has been shown to be responsible for the specific processing of β-oxidation enzymes in the peroxisomal matrix (e.g., the removal of leader peptide of 3-ketoacyl-CoA thiolase A) (Kurochkin et al., 2007; Mizuno et al., 2013). Interestingly, a recent study has shown that the proteolytic activities of Tysnd1 and LONP2 cooperatively regulate peroxisomal fatty acid β-oxidation (Okumoto et al., 2011). Taken together, these findings clearly show that mammalian peroxisomes contain a highly sophisticated protease-dependent house-keeping system to ensure protein quality within the organellar matrix.

Some time ago, it was demonstrated that the turnover rates of some PMPs (e.g., Pex3p and Pex16p) are much faster than that of matrix proteins (Matsuzaki and Fujiki, 2008; Huybrechts et al., 2009), and that the half-life of these PMPs can be extended by inhibiting the ubiquitin-proteasome system (UPS) (Huybrechts et al., 2009). These observations indicate that the peroxisomal membrane continuously undergoes quality control mechanisms in order to remove unnecessary or dysfunctional membrane proteins (Figure 1). Unfortunately, the mechanisms underlying this process remain unclear. However, in this context, it is necessary to mention that—as part of a quality control mechanism—membrane-associated PTS (co-)receptors (e.g., Pex5p, Pex7p, and Pex20p) also can be degraded by the UPS, at least in several organisms (Figure 1) (Léon et al., 2006; Cui et al., 2013). Under normal conditions, Pex5p and Pex20p become mono-ubiquitinated at a conserved cysteine residue. This triggers the subsequent ATP-dependent dislocation of these receptors from the peroxisomal membrane back to the cytosol where they become available for a new import cycle (Grou et al., 2009). However, under conditions where export of these receptors is impaired, these peroxins are polyubiquitinated on one or more lysines in their N-terminal tails and extracted from the peroxisomal membrane for degradation by the UPS by a process called RADAR (for Receptor Accumulation and Degradation in the Absence of Recycling) (Léon et al., 2006). As RADAR closely resembles ERAD (for Endoplasmic Reticulum-Associated protein Degradation) (Gabaldón et al., 2006; Schlüter et al., 2006), a pathway in which defective proteins in the ER are exported back to the cytosol for proteasomal degradation (Brodsky, 2012), it is tempting to speculate that peroxisomal matrix proteins also may exit the organelle for cytosolic degradation (Figure 1). This hypothesis is in line with the recent observation that, in plants, the efficient degradation of peroxisomal matrix proteins involves Pex6p, an AAA (ATPase Associated with various cellular Activities)-ATPase that is part of the Pex5p export machinery (Burkhart et al., 2013). Finally, it has been reported that in plant cells the degradation of peroxisome-associated Pex7p is triggered by binding to RabE1c, a small Ras-related GTPase (Cui et al., 2013). Note that, as Pex5p levels are drastically reduced in skin fibroblasts from PBD patients that are blocked in normal receptor recycling (e.g., cells lacking Pex1p or Pex6p activity) (Dodt and Gould, 1996), the RADAR quality control pathway is most likely also conserved in mammals (Figure 1). So far, there is no evidence for a UPS-mediated degradation mechanism of mammalian Pex7p.

## Peroxisome degradation

To maintain a healthy cellular peroxisome population, dysfunctional and superfluous organelles need to be selectively removed. Throughout the years, several half-life studies on peroxisomal proteins have been performed, and—if one assumes that peroxisomes are degraded as a whole—most data indicate a peroxisomal half-life of around 1.5–2 days (Price et al., 1962; Poole et al., 1969; Huybrechts et al., 2009). This turnover process may occur randomly (e.g., as part of bulk sequestration of the cytoplasm) or selectively. However, one must assume that a cell—in order to assure a functional peroxisome population—preferably and specifically degrades non-functional organelles. Below we discuss the concepts of peroxisome removal, the initial triggers for peroxisomal degradation, and the factors for dysfunctional peroxisome recognition.

### Concepts of peroxisome removal

Until now, ample evidence has been provided that peroxisomes are mainly degraded by the autophagy-lysosome pathway, in a process known as pexophagy (see below). In addition, it has been suggested that these organelles can be targets for 15-lipoxygenase (15-LOX)-mediated autolysis. Both degradation pathways are discussed in the following paragraphs.

Autophagy is a highly conserved intracellular pathway that delivers cytoplasmic substrates to lysosomes for subsequent degradation (Choi et al., 2013). Under basal conditions, this process provides a mechanism for the removal of long-lived proteins and the turnover of superfluous and damaged organelles (Mizushima et al., 2011). However, this degradation pathway can also be upregulated in response to different stress conditions such as hypoxia, heat, and starvation. Yeast genetics has been crucial for the elucidation of the molecular machinery responsible for autophagy, and, to date, 36 AuTophaGy-related (*ATG*) genes have been identified (Motley et al., 2012). Of these, many are part of the core autophagy machinery essential for the formation of canonical autophagosomes (see below), whereas others function only in different selective autophagy pathways (Mizushima et al., 2011). For more details regarding the molecular mechanisms of autophagy, we refer the reader to other excellent reviews (Klionsky et al., 2011; Mizushima et al., 2011).

Until now, three major types of autophagy have been characterized in eukaryotic cells: macroautophagy, microautophagy, and chaperone-mediated autophagy (CMA). During macroautophagy, parts of the cytoplasm are engulfed within double- or multi-membrane delimited structures known as autophagosomes, which subsequently fuse with lysosomes where cargo is released (Mizushima et al., 2011). In contrast, microautophagy involves the direct engulfment of cytoplasmic portions at the lysosome by invagination, protrusion or septation of the lysosomal membrane (Chen and Klionsky, 2011). Finally, CMA is dependent on chaperones which selectively target cytosolic proteins containing a pentapeptide motif (KFERQ) to the lysosomal surface, where the protein is unfolded and transported across the membrane (Kaushik and Cuervo, 2012). Since CMA only degrades cytosolic proteins (Kaushik and Cuervo, 2012) and selective organellar microautophagy has not been unambiguously proven to take place in mammals (Mijaljica et al., 2011), macroautophagy is widely believed to be the major, if not only, pathway for organelle degradation in mammalian cells.

In 1966, de Duve and Baudhuin were the first scientists to discuss the occasional appearance of peroxisomes within autophagosomes, but thought that lysosomal degradation by itself was insufficient to account for the high cellular turnover of catalase (de Duve and Baudhuin, 1966). Since then, several studies on cultured cells have shown that in the presence of 3-methyladenine (3-MA), a macroautophagic inhibitor, peroxisome degradation is strongly inhibited (Luiken et al., 1992; Kondo and Makita, 1997; Huybrechts et al., 2009). After the discovery of peroxisome proliferators (Reddy et al., 1980), a new method became available to study the degradation of superfluous peroxisomes in rodents. As already mentioned above (see section Regulation of Peroxisome Abundance), treatment of these animals with hypolipidemic drugs massively increases the number of peroxisomes, which—after removal of stimulus—rapidly returns to basal levels. However, the mechanism by which peroxisomes disappear remained enigmatic until 1993, when excess peroxisomes were detected within autophagosomes and lysosomes upon addition of the lysosomal protease inhibitor leupeptin (Yokota, 1993). More recently, these observations were confirmed and extended to be macroautophagy-dependent (Iwata et al., 2006). This conclusion was based on the observation that degradation of proliferated peroxisomes was impaired in autophagy-deficient (*Atg7*^−/−^) mouse hepatocytes (Iwata et al., 2006). Nevertheless, as peroxisome abundance still slightly decreased upon withdrawal of the proliferation stimulus, peroxisomes can most likely also be degraded by other mechanisms.

Another mechanism proposed to play a role in peroxisome degradation is 15-LOX-dependent membrane autolysis (Figure 1B). Lipoxygenases are a family of monomeric non-heme, non-sulfur iron dioxygenases, which catalyze the conversion of poly-unsaturated fatty acids (PUFAs) into conjugated hydroperoxides (Maccarrone et al., 2001). The actions of 15-LOX are thought to be important for organellar degradation in reticulocytes, central fiber cells of the eye lens, and keratinocytes (van Leyen et al., 1998). In these cells, the expression of 15-LOX peaks just before organellar degradation occurs (van Leyen et al., 1998). The potential role of 15-LOX has been strengthened by the observation that organellar degradation in these cells occurs independently of autophagy (Matsui et al., 2006), although this is an issue under debate (Betin et al., 2013). About a decade ago, it was shown that—in rat liver—peroxisomal membranes were disrupted when cells were fixed in medium conserving 15-LOX activity (Yokota et al., 2001). This process was effectively blocked upon addition of the 15-LOX inhibitors esculetin and propyl gallate (Yokota et al., 2001). In addition, it is important to note that 15-LOX was shown to colocalize with some, albeit not all peroxisomes (Yokota et al., 2001). Taken together, these finding suggest that peroxisomes, depending on the cell type and/or their membrane lipid composition, may be targets for 15-LOX-mediated autolysis.

Finally, one cannot rule out that in certain cell types and/or under specific environmental conditions, peroxisome degradation may occur through other mechanisms. In this context, it is interesting to note that (1) there is some experimental evidence that cell organelles may also be degraded by non-conventional Atg5/Atg7-independent autophagy pathways (Nishida et al., 2009; Juenemann and Reits, 2012), and (2) inhibition of cellular respiration and uncoupling of oxidative phosphorylation in HeLa cells resulted in the selective elimination of dysfunctional mitochondria by a novel mechanism involving the formation of “mitoptotic bodies,” which are subsequently extruded from the cells (Lyamzaev et al., 2008).

### Recognition factors and adaptor proteins for peroxisome removal

Mounting evidence suggests that autophagy is a more selective process than originally anticipated. Most of the pioneering studies on pexophagy have been done using methylotrophic yeasts as the model organism. Working with these yeasts has several advantages, including the relative ease by which peroxisome number, volume and content can be modulated by shifts in growth medium, and the fact that it is rather straightforward to genetically modify these organisms (Manjithaya et al., 2010). Below, we therefore include data from different organisms in order to get a clearer picture of mammalian pexophagy. Until now, every selective autophagy pathway requires the involvement of specific cargo receptors (Till et al., 2012). These receptors, which act independently or together with specific adaptor proteins, recognize their substrates and connect them with one or more components of the core autophagic machinery to allow their specific sequestration (Johansen and Lamark, 2011). To date, at least five autophagic receptors have been identified in mammals: p62, NDP52, optineurin, NIX, and NBR1 (Behrends and Fulda, 2012). These receptors work alone or co-operatively in targeting their substrates for selective degradation (Johansen and Lamark, 2011). The modular composition of binding domains and motifs in these receptors ensures efficient tethering of cargo to the site of developing and engulfing autophagosomes (Behrends and Fulda, 2012). Common for most of these receptors is that they contain both an LC3-Interacting Region (LIR) and a Ubiquitin-Binding Domain (UBD) (Behrends and Fulda, 2012). LC3 and its homologues GABARAP and GATE-16 are ubiquitin-like proteins that are synthesized as precursors and—upon autophagy induction—processed and localized to the autophagosomal membranes (Mizushima et al., 2011). The LIR and UBD domains render the adaptors capable of bridging a ubiquitinated substrate (e.g., organelles, protein aggregates, and bacteria) with the autophagic machinery, thereby selectively triggering degradation of the cargo.

Until now, at least three pexophagy receptors have been identified, including Atg30 (for *P. pastoris* and related yeasts) (Farré et al., 2008), Atg36 (for *Saccharomyces cerevisiae* and similar yeasts) (Motley et al., 2012) and NBR1 and/or p62 (for mammalian cells) (Kim et al., 2008; Deosaran et al., 2013). These proteins bridge peroxisomes with developing autophagosomes by simultaneously binding to protein(s) at the peroxisomal membrane and the autophagic machinery via different structural motifs (Till et al., 2012). The *P. pastoris* peroxisome receptor Atg30 interacts with peroxisomes through two PMPs, Pex3p, and Pex14p, and with the autophagic machinery via Atg11 and Atg17 (Farré et al., 2008). *S. cerevisiae* Atg36, another Atg11-interacting protein, is also recruited to peroxisomes in a Pex3p-dependent manner (Motley et al., 2012). Interestingly, both Atg30 and Atg36 are regulated by phosphorylation (Farré et al., 2013), trigger pexophagy upon overexpression (Farré et al., 2008; Motley et al., 2012), and interact with Atg11 (Farré et al., 2008; Motley et al., 2012). Atg11 is thought to function as a common adaptor protein for most, if not all, selective autophagy pathways in yeasts (Manjithaya et al., 2010). Note that, despite their functional similarities, Atg30 and Atg36 do not display any sequence homology (van der Zand and Reggiori, 2012).

Less is known about the selective pexophagy receptors in mammals. However, some years ago, it was discovered that peroxisomes can be degraded in a p62-dependent manner upon overexpression of a PMP (in this case PMP34 and Pex3p) fused to a cytosol-facing ubiquitin moiety (Figure 1B) (Kim et al., 2008). This phenotype can be significantly increased by employing a mutated ubiquitin protein incapable of being poly-ubiquitinated, thus eliminating proteasome-dependent removal of the proteins from the peroxisomal membrane (Kim et al., 2008). In addition, a recent study showed that pexophagy was triggered upon overexpression of NBR1, another adaptor protein (Figure 1B) (Deosaran et al., 2013). However, similar overexpression of p62 did not yield the same results, indicating that NBR1 most likely functions as endogenous pexophagy adaptor in mammals (Deosaran et al., 2013). This might stem from the fact that, even though these proteins share LIR and UBD domains, NBR1—but not p62—contains a membrane interacting amphipathic α-helical JUBA domain, capable of binding to the peroxisomal lipid bilayer (Deosaran et al., 2013). Nevertheless, it cannot be excluded that these proteins co-operate during pexophagy. Finally, it should be mentioned that, unlike Atg30 and Atg36, both p62, and NBR1 have been implicated in the selective degradation of other cargoes (Johansen and Lamark, 2011). Taken together, these data clearly indicate that mammalian pexophagy is regulated by at least one, and perhaps even more, of the currently identified autophagy receptors. An intriguing question that has risen from these studies is how these receptors recognize peroxisomes as their targets.

### Peroxisomal components necessary for pexophagy

Currently, most data point to a role of Pex3p and/or Pex14p in the recruitment of pexophagy-specific receptor proteins. For example, while in *H. polymorpha* peroxisome degradation is triggered by the removal of Pex3p (Bellu et al., 2002; van Zutphen et al., 2011), studies in *P. pastoris* and *S. cerevisiae* have shown that this peroxin is essential to recruit Atg30 and Atg36, respectively, to the peroxisome prior to degradation (Farré et al., 2008; Motley et al., 2012). In addition, *P. pastoris* Atg30 has been reported to interact with Pex14p (Farré et al., 2008), and the N-terminal 64 amino acids of this peroxin are required for pexophagy in *H. polymorpha* (Bellu et al., 2001; van Zutphen et al., 2008). Interestingly, by redirecting Pex3p to the mitochondrial outer membrane in yeast cells lacking Atg32, the mitochondria-specific autophagy receptor (Okamoto et al., 2009), it is possible to recruit Atg36 to this organelle and trigger mitophagy (Motley et al., 2012).

In mammals, there is some evidence that Pex14p may play a role in pexophagy by interacting with LC3-II during nutrient-starvation (Figure 1B) (Hara-Kuge and Fujiki, 2008). In addition, a recent study by Deosaran and colleagues suggests that (mono)-ubiquitination of endogenous PMPs can trigger pexophagy (Deosaran et al., 2013). Unfortunately, no such protein has yet been identified. One potential candidate is Pex5p, which needs to be mono-ubiquitinated at the peroxisomal membrane in order to be recycled back to the cytosol (Platta et al., 2013). Indeed it has recently been observed that by inhibiting Pex5p recruitment to peroxisomes via down-regulation of Pex14p, pexophagy is—at least partly—prevented upon overexpression of NBR1 (Deosaran et al., 2013). However, since (1) Pex14p is heavily implicated in yeast pexophagy (Till et al., 2012; see above), and (2) this peroxin interacts with membrane-bound LC3-II during starvation conditions (Hara-Kuge and Fujiki, 2008), one cannot assertively claim that the lack of peroxisome turnover was due to the absence of Pex5p, and not to Pex14p (or any other Pex14p-interacting factor), at the peroxisomal membrane.

### Triggers for peroxisome degradation

Although relatively much is known about the concepts and recognition factors of peroxisome degradation, less data exist regarding the triggers for this process. As mentioned before, both superfluous and dysfunctional organelles need to be removed in order to maintain cellular homeostasis. The turnover of superfluous peroxisomes can be induced by returning to growth conditions in which the necessity of peroxisomes is reduced (see section Regulation of Peroxisome Abundance). In addition, overexpression of the pexophagy receptors Atg30, Atg36, and NBR1 has been shown to trigger peroxisome degradation by binding simultaneously to peroxisomes and the autophagic machinery (see section Recognition Factors and Adaptor Proteins for Peroxisome Removal). Accumulating evidence indicates that the initial signal for peroxisome degradation resides at the peroxisomal membrane, and that changes in its composition may be the key for pexophagy induction. This gives rise to a burning question in the field: how is, at a given time point, a select set of peroxisomes recognized by the autophagic machinery whereas others are not? A potential answer to this question could reside in the existence of peroxisomal subpopulations, where some peroxisomes are protected from degradation. In this context, it is important to mention that, in yeasts, at least one peroxisome is protected from degradation under pexophagy-inducing conditions (Leao-Helder et al., 2003). In addition, even high overexpression of pexophagic receptors does not yield a total cellular lack of peroxisomes (Farré et al., 2008; Motley et al., 2012; Deosaran et al., 2013). Furthermore, it is likely that organelle morphology may also play a role. For example, it has been shown that—during starvation-induced autophagy—mitochondria elongate and are therefore protected from mitophagy (Gomes et al., 2011). Since (1) peroxisomal morphogenesis is a very dynamic process (Ribeiro et al., 2012), and (2) peroxisomes are commonly elongated during proliferation (Schrader et al., 2012), one could envisage a similar protective mechanism for these organelles. In this context, it is worthwhile mentioning that studies in *P. pastoris* have shown that the larger the peroxisome, the more cargo-specific Atg proteins are essential for its sequestration (Nazarko et al., 2009).

Other essential questions that need to be addressed include the identity and order of events that lead to the substrate recognition signal at the peroxisomal membrane. In this context, one can envisage that the signal for peroxisome degradation stems from the peroxisomal matrix. Indeed, as (1) peroxisomes are important regulators of both ROS and lipid metabolism (Van Veldhoven, 2010; Fransen et al., 2012), and (2) it has been shown that inhibition of autophagy with 3-MA increased the amount of peroxisomes with a disturbed redox state (Ivashchenko et al., 2011), it is tempting to speculate that a disrupted redox equilibrium can lead to oxidation-specific peroxisomal membrane modifications, such as lipid peroxidation. Here it is also important to mention that mitochondria are depolarized and subjected to mitophagy upon compartment-specific ROS-generation (Kim and Lemasters, 2011; Wang et al., 2012). However, since peroxisomes do not contain a membrane potential, a similar mechanism seems unlikely to occur for these organelles. Nevertheless, as (1) it is well-known that the PTEN Induced Putative Kinase 1 (PINK1)-dependent recruitment of the E3 ubiquitin ligase Parkin to the mitochondrial outer membrane can trigger mitophagy (Lazarou et al., 2012; Ashrafi and Schwarz, 2013), and (2) targeting ectopically expressed PINK1 to the peroxisomal membrane recruits Parkin to these organelles and triggers pexophagy (Lazarou et al., 2012), it is very likely that the peroxisome- and mitochondria-specific turnover mechanisms converge at an early step. This hypothesis is also in line with the observation that mitochondria-targeted Pex3p triggers mitophagy in *H. polymorpha* mutants lacking Atg32 (see section Peroxisomal Components Necessary for Pexophagy) (Motley et al., 2012).

It is widely known that mitochondria harbor complex fusion and fission machineries, which allow them to mix, segregate and eliminate damaged components from the functional networking population (Twig et al., 2008). In addition, there is abundant evidence that mitochondrial dynamics and mitophagy are closely related, and that a dysfunctional mitochondrion has to be separated from the mitochondrial network before it can be sequestered by an autophagosome (Ashrafi and Schwarz, 2013). However, as peroxisomes cannot fuse with one another (Huybrechts et al., 2009; Bonekamp et al., 2012), alternative mechanisms must assist in assuring a healthy organelle population. One such mechanism may be asymmetric fission. This would render peroxisomes capable of sequestering non-functional proteins into the mother organelle, which—after a limited number of fission events—is targeted for pexophagy (Huybrechts et al., 2009; Delille et al., 2010). In this context, it is important to mention that, in *H. polymorpha*, protein aggregates within the peroxisomal lumen can be eliminated by the concerted action of asymmetric fission and subsequent autophagic degradation of the aggregate-containing organelle (Manivannan et al., 2013).

## Physiological role of pexophagy

As already mentioned above (see section Physiological Importance of Peroxisome Homeostasis), peroxisomes play a prominent role in a variety of cellular metabolic and signaling processes. As such, a tight regulation of peroxisome biogenesis, dynamics, and degradation is important for human health. Over the years, it has become increasingly clear that not only defects in peroxisome biogenesis, but also disturbances in peroxisome degradation can contribute to disease (see section Physiological Importance of Peroxisome Homeostasis). This is illustrated below by three specific examples.

First, there is accumulating evidence that defects in pexophagy can facilitate the cellular aging process. For example, it is already known for more than a decade that the number of peroxisomes profoundly increases during cellular aging, and that these organelles display a reduced capacity to import matrix proteins, especially catalase (Legakis et al., 2002). Since (1) these cells contain peroxisomes with a disturbed H_2_O_2_ metabolism (Legakis et al., 2002), (2) there is strong evidence that oxidative stress plays a key role in the etiology and progression of cellular senescence (Salmon et al., 2010), and (3) the latter process is causally linked to organismal aging (Baker et al., 2011), it is very likely that these dysfunctional peroxisomes directly contribute to the age-related phenotype (Koepke et al., 2008). As these age-related changes in peroxisome number, matrix protein import, and ROS production can be mimicked in a *H. polymorpha* strain lacking Atg1, a crucial member of the core autophagic machinery (Aksam et al., 2007), these phenotypes in all probability result from an age-related decline in lysosomal function or pexophagy-specific factors.

Next, it has also been postulated that pexophagy is essential to maintain functional peroxisomes during endotoxin-induced stress (Vasko et al., 2013). In this study, it was shown that exposure of human vascular endothelial cells or mice to lipopolysaccharides (LPS) selectively induced pexophagy, and that inhibition of this process (e.g., by treating the cells with chloroquine or by employing lysosome-defective Lyst-mice) resulted in the accumulation of functionally compromised peroxisomes, an altered cellular redox equilibrium, and aggravated renal damage.

Finally, as it is well-known that the decreased autophagic flux observed in various lysosomal storage diseases (LSDs) often leads to an accumulation of dysfunctional mitochondria and cytoplasmic protein aggregates (Platt et al., 2012), the same is most likely true for peroxisomes. LSDs are a family of genetic disorders that perturb lysosomal homeostasis by the accumulation of specific macromolecules or monomeric catabolic products inside organelles of the endosomal-autophagic-lysosomal system (Platt et al., 2012). Interestingly, some LSDs such as Niemann-Pick disease type 1 (Schedin et al., 1997) and Krabbe disease (Haq et al., 2006) have also been associated with peroxisome dysfunction. In addition, as (1) a-series gangliosides and their precursor are common secondary storage metabolites in many LSDs, and (2) these compounds also increase in PBDs, it is very likely that peroxisomal dysfunction underpins secondary ganglioside storage in LSDs (Platt et al., 2012). Taken together, these data indicate that a disturbance in pexophagy may have a significant negative impact on human health and function.

## Conclusions and perspectives

From the cumulative evidence presented in this review, it is clear that macroautophagy plays a pivotal role in the removal of obsolete peroxisomes in mammalian cells. However, as (1) these organelles can also be degraded under conditions where the conventional macroautophagy pathway is inactivated (Iwata et al., 2006), and (2) macroautophagy does not seem to be responsible for organelle turnover during lens and erythroid differentiation (Matsui et al., 2006), it is very probable that other cell- and/or condition-specific peroxisome degradation pathways exist. Candidate pathways may include micropexophagy, Atg5/Atg7-independent macropexophagy, and 15-LOX-mediated degradation. Importantly, crucial *in vivo* evidence for the presence of these or other peroxisome turnover routes is currently lacking. Yet, the recent identification of NBR1 as potential pexophagy receptor may shed more light on this issue (Deosaran et al., 2013). However, given the functional similarities of mammalian autophagy receptors (Behrends and Fulda, 2012), it is likely that by inactivating NBR1 (e.g., in cells or in an animal model) other autophagic receptors may shoulder the role of this protein. In addition, as NBR1 has been shown to be involved in other ubiquitin-regulated autophagy pathways (Kirkin et al., 2009), the phenotype observed upon NBR1 inactivation will not be solely due to impaired peroxisome degradation.

Virtually all experimental data suggest that, at least in yeasts, peroxisome degradation is a highly selective process (Manjithaya et al., 2010; Till et al., 2012). Although not yet unambiguously proven, several arguments support the view that this is also true for mammals. For example, peroxisomes induced by PPARα-agonists are selectively removed upon withdrawal of the proliferation stimulus (Yokota, 1993). In addition, although Pex14p has been shown to interact with LC3-II during nutrient starvation, peroxisome degradation only occurred when the cells were re-cultured in a normal medium (Hara-Kuge and Fujiki, 2008). These data are in line with the finding that starvation-induced autophagy of cell organelles occurs in an ordered fashion (Kristensen et al., 2008).

The observation that, in yeasts, peroxisome biogenesis and degradation converge on Pex3p and Pex14p offers the intriguing possibility that these peroxins may act as peroxisome fate decision makers. Whether this is also the case in mammals remains to be established. In this context, it is important to mention that (1) mammalian Pex14p can directly bind to Pex5p and LC3-II, and (2) the affinity of Pex14p for these proteins depends on the culture conditions (with Pex5p and LC3-II being the preferred interaction partner under nutrient-rich and starvation conditions, respectively) (Figure 1B) (Hara-Kuge and Fujiki, 2008). Interestingly, Pex14p is also capable of forming distinct oligomeric complexes at the peroxisomal membrane (Itoh and Fujiki, 2006). As the functions of these complexes are not yet characterized, it is tempting to speculate that some may fulfill a specific role in peroxisome turnover.

One of the most challenging aspects within the field is the identification of potential triggers for peroxisome degradation. As (1) excessive ROS-generation in mitochondria has been shown to trigger mitophagy (Kim and Lemasters, 2011; Wang et al., 2012), and (2) peroxisomes produce large amounts of ROS as part of their normal metabolism (Fransen et al., 2012), it is very likely that also disturbances in peroxisomal redox metabolism may provoke signaling/damaging events that lead to structural changes in the peroxisomal membrane and ultimately result in organelle degradation. One such modification may be lipid peroxidation. Alternatively, the initial trigger may be generated by changes in peroxisomal lipid metabolism, a condition likely to affect the organellar membrane composition. In this context, it is worthwhile mentioning that (1) DHA, a PUFA synthesized by peroxisomal β-oxidation (Van Veldhoven, 2010), can promote negative membrane curvature (Bruno et al., 2007), and (2) NBR1, the putative pexophagy receptor in mammalian cells, contains a lipid binding domain that inserts into the peroxisomal membrane bilayer in a curvature-dependent manner (Mardakheh et al., 2010; Deosaran et al., 2013). These findings and the observation that DHA can also mediate peroxisome elongation (Itoyama et al., 2012) suggest that peroxisomal β-oxidation, peroxisome morphology, and pexophagy are closely intertwined processes. Importantly, such a mechanism would closely resemble that of mitochondria in that dysfunctional spherical organelles are segregated from a tubular network prior to degradation (Ashrafi and Schwarz, 2013).

Finally, it is very likely that the implications of dysfunctional peroxisome degradation have been overlooked throughout the years. In this context, it is essential to take into account that an increase in peroxisome number (e.g., during cellular aging) is not necessarily due to an augmentation of peroxisome biogenesis, but can also result from a decrease in peroxisome turnover rates. Unfortunately, with the current lack of animal models that are selectively deficient in peroxisome degradation, it is virtually impossible to predict the severity of the phenotype and/or the course of the disease of patients suffering from this impairment. However, given the recent breakthroughs in this field, we are convinced that such disease models will soon be available, and answers to these important questions rapidly obtained.

### Conflict of interest statement

The authors declare that the research was conducted in the absence of any commercial or financial relationships that could be construed as a potential conflict of interest.
